# Modification of genetic regulation of a heterologous chitosanase gene in *Streptomyces lividans *TK24 leads to chitosanase production in the absence of chitosan

**DOI:** 10.1186/1475-2859-10-7

**Published:** 2011-02-10

**Authors:** Marie-Pierre Dubeau, Isabelle Guay, Ryszard Brzezinski

**Affiliations:** 1 Centre d'Étude et de Valorisation de la Diversité Microbienne, Département de Biologie, Faculté des Sciences, Université de Sherbrooke, 2500 boulevard de l'Université, Sherbrooke, J1K 2R1, (Québec) Canada

## Abstract

**Background:**

Chitosanases are enzymes hydrolysing chitosan, a β-1,4 linked D-glucosamine bio-polymer. Chitosan oligosaccharides have numerous emerging applications and chitosanases can be used for industrial enzymatic hydrolysis of chitosan. These extracellular enzymes, produced by many organisms including fungi and bacteria, are well studied at the biochemical and enzymatic level but very few works were dedicated to the regulation of their gene expression. This is the first study on the genetic regulation of a heterologous chitosanase gene (*csnN106*) in *Streptomyces lividans*.

**Results:**

Two *S. lividans *strains were used for induction experiments: the wild type strain and its mutant (*ΔcsnR)*, harbouring an in-frame deletion of the *csnR *gene, encoding a negative transcriptional regulator. Comparison of chitosanase levels in various media indicated that CsnR regulates negatively the expression of the heterologous chitosanase gene *csnN106*. Using the *ΔcsnR *host and a mutated *csnN106 *gene with a modified transcription operator, substantial levels of chitosanase could be produced in the absence of chitosan, using inexpensive medium components. Furthermore, chitosanase production was of higher quality as lower levels of extracellular protease and protein contaminants were observed.

**Conclusions:**

This new chitosanase production system is of interest for biotechnology as only common media components are used and enzyme of high degree of purity is obtained directly in the culture supernatant.

## Background

Chitosan, a partly *N*-deacetylated form of chitin, is naturally found in the cell walls of fungi, especially in *Zygomycetes *(*Mucor *sp., *Rhizopus *sp.), and in the green algae *Chlorophyceae *(*Chlorella *sp.) [1-3]. Chitosan, is a polysaccharide made of β-1,4-linked D-glucosamine (GlcN) units with a variable content of *N*-acetyl-D-glucosamine units. Chitosan is produced at industrial scale by alkaline deacetylation of chitin, originating mainly from crustacean shells [4]. This polysaccharide, almost unique among natural polymers for its amino groups that remain positively charged in mild acidic solutions, is the subject of numerous works oriented towards its numerous emerging applications in medicine, agriculture, dietetics, environment protection and several other fields [5-7]. Chitosan is also a valuable source of GlcN, a neutraceutical used as a therapeutic agent in osteoarthritis [8]. Many properties of chitosan, especially in biological applications are dependent on its molecular weight, i.e. on its degree of polymerization. The very short derivatives of chitosan - the chito-oligosaccharides are of particular interest, due to their increased solubility in aqueous solutions and their specific biological activities [9,10].

To obtain chitosan chain of varying degrees of polymerization, several chemical and physical techniques were investigated [11-13]. Enzymatic techniques with either free or immobilized chitinase or chitosanase enzymes are also intensively studied [14-16]. Chitosanase production has been found in many microorganisms, bacteria or fungi. The enzymes so far characterized at the primary sequence level belong to seven families of glycoside hydrolases: GH3, GH5, GH7, GH8, GH46, GH75 and GH80 [17-24]. While these enzymes are *endo*-hydrolases, their mechanism could potentially be transformed into *exo*-type by protein engineering as shown for the GH46 chitosanase from *Bacillus circulans *MH-K1 [25]. Chitosan can be also hydrolyzed by enzymes acting by an exo-mechanism generating GlcN monomers [26,27]. The chitosanases from *Streptomyces *have been widely studied in various aspects of structure-function relationships [28 and references cited herein]. Usually, these chitosanases are produced in the heterologous host *Streptomyces lividans via *the multi-copy vector pFD666 [29]. However, very few works have been dedicated to the regulation of chitosanase gene expression in the native and/or heterologous hosts. Most studies were limited to the follow up of chitosanase production in various culture media [30,31].

The present report is the first study dedicated to the optimization of gene expression of a chitosanase in a heterologous host. The chitosanase gene under study, *csnN106 *has been cloned from the *Kitasatospora sp*. N106 strain (formerly *Nocardioides *sp. N106) [32]. The chitosanase CsnN106 is highly similar to other GH46 family chitosanases at the structural and biochemical level [33]. The strain N106 was among the most active chitosanolytic strains isolated through an extensive screening of soil samples [32,34].

In our previous work we observed that an efficient production of CsnN106 chitosanase in *Streptomyces lividans *TK24 was strictly dependent on the addition of chitosan or its derivatives to the culture medium [35] indicating that this foreign gene is still subjected to some kind of chitosan-dependent regulation in the heterologous host. However, the addition of chitosan as a component in any culture medium is not without problems due to the well known anti-microbial properties of this polysaccharide [9,10] which can slow down the bacterial growth.

Here, we show that the expression of the heterologous gene *csnN106 *in *S. lividans *is regulated at the transcriptional level. This led us to engineer a new expression system which does not require anymore the presence of chitosan or its derivatives as inducers of enzyme production.

## Results

### The rationale of genetic constructions

The integrative plasmid pHM8aBΔM [36,37], was used in studies involving the regulation of gene *csnN106 *expression. The *csnN106 *gene was present in a single copy in the genome, avoiding the regulatory interference brought by multi-copy plasmids.

**Figure 1:**
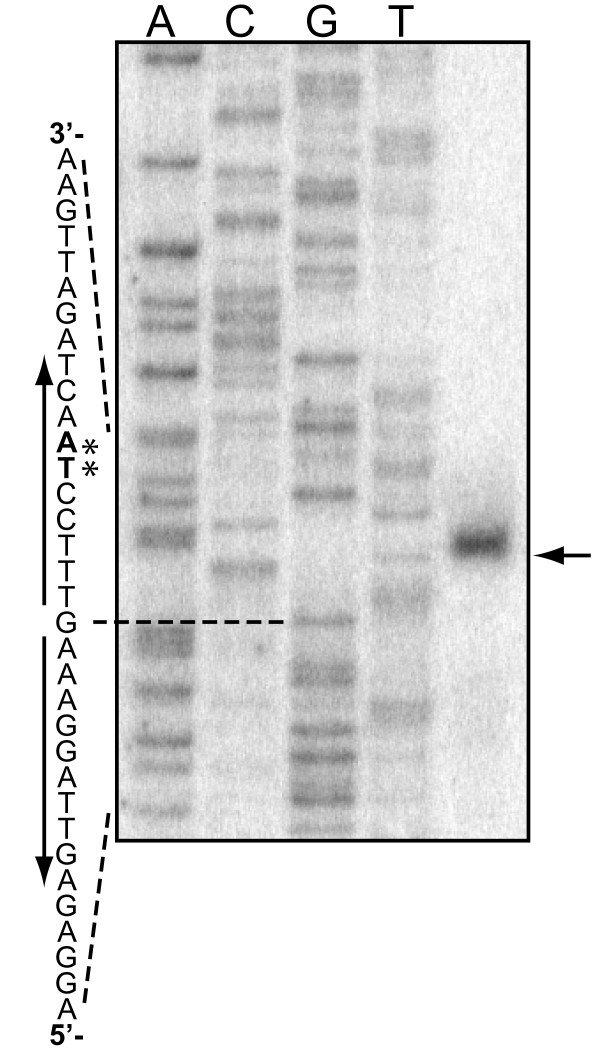
**Primer extension analysis of *csnN106 *transcripts**. The apparent 5' terminus for the *csnN106 *transcript was identified by annealing a radiolabeled primer complementary to the mRNA of *csnN106 *and extension with reverse transcriptase. 40 μg of total RNA, from GlcN-chitosan oligomers induced *S. lividans *TK24(pHPr-WT), were used for extension reaction. The same primer was used for DNA sequencing reactions with the pHPr-WT plasmid. (→): primer extension product; (*): apparent transcription start site. Vertical arrows: palindromic sequence.

**Figure 2:**
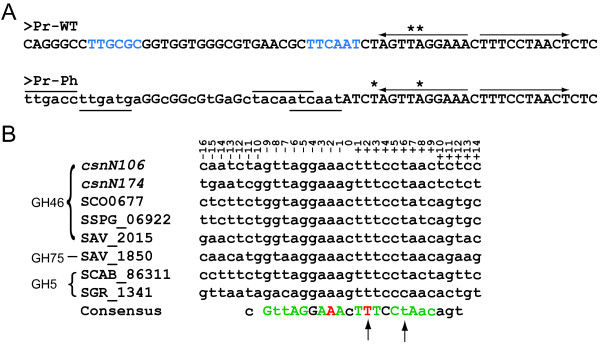
**Promoter regions characterized in this work**. **(A) **Fragment of the promoter region of *csnN106 *gene variants. Pr-WT: native promoter region, the putative -35 and -10 boxes are indicated in blue. Pr-Ph: a construct in which the native promoter has been replaced by a double promoter from *Streptomyces ghanaensis *phage I19, the respective -35 and -10 boxes are over and underlined. Low case letters indicate nucleotide changes between Pr-WT and Pr-PH. (*): start points of transcription. Arrows: inverted repeats of the palindromic box. **(B) **Alignment of palindromic sequences present in the promoter regions of chitosanase genes in actinomycetes. Nucleotides are numbered relative to the center of symmetry. In the consensus sequence, nucleotides are coloured according to their importance for DNA-protein interaction established by equilibrium competition experiments [39]: red: nucleotides critical for interaction; green: nucleotides moderately important for interaction; black: nucleotides without apparent effect on interaction. (↑): base pairs mutated in the Pr-Pa construct. GH: glycoside hydrolase family.

By primer extension, we determined the start site for mRNA transcribed from *csnN106 *(Figure 1 and Figure 2A), defining the probable -35 and -10 boxes of the promoter of *csnN106 *as TTGCGC and TTCAAT with a spacer of 18 nucleotides (shown in blue on Figure 2A). To test another promoter, described as a "strong" promoter by Labes *et al*. [38], the original -35 and -10 boxes of *csnN106 *gene were substituted with the two tandemly arrayed and overlapping promoters of the *Streptomyces ghanaensis *phage I19, taking the respective transcription start sites as reference (Figure 2A).

A palindromic sequence overlaps the transcriptional start site of *csnN106 *(Figure 2A). Highly similar sequences are also present upstream from the coding sequences of chitosanase genes found in other genomes of actinomycetes, displaying a clear consensus (Figure 2B). Previous gel retardation experiments have shown an interaction between a protein present in partially purified cell extract from *Kitasatospora *sp. N106 and a short DNA segment including the palindromic sequence [39]. Competition tests with mutated oligonucleotides allowed determining the bases which were critical for the interaction with the regulatory protein in vitro (Figure 2B) [39]. For the present study, two most important base pairs in the right half of the palindromic sequence were mutated (while keeping intact the original -10 and -35 promoter boxes) and introduced upstream from the *csnN106 *coding sequence, resulting in a third version of this heterologous gene. These three genes were introduced in two hosts: *Streptomyces lividans *TK24 (the host used so far in most works involving actinobacterial chitosanase studies) and a mutant harbouring an in-frame deletion in *csnR *gene (*ΔcsnR*, formerly described as *Δ2657 h *by Dubeau *et al*. [37]). The *csnR *gene (SSPG_04872, according to GenBank annotation) is coding for the transcriptional regulator of the endogenous chitosanase gene (Dubeau, M.-P., Poulin-Laprade, D., Ghinet, M. G., Brzezinski, R.: Characterization of CsnR, the transcriptional repressor of the chitosanase gene of *Streptomyces lividans*, submitted), a protein belonging to the ROK family created by Titgemeyer *et al*. [40].

**Figure 3:**
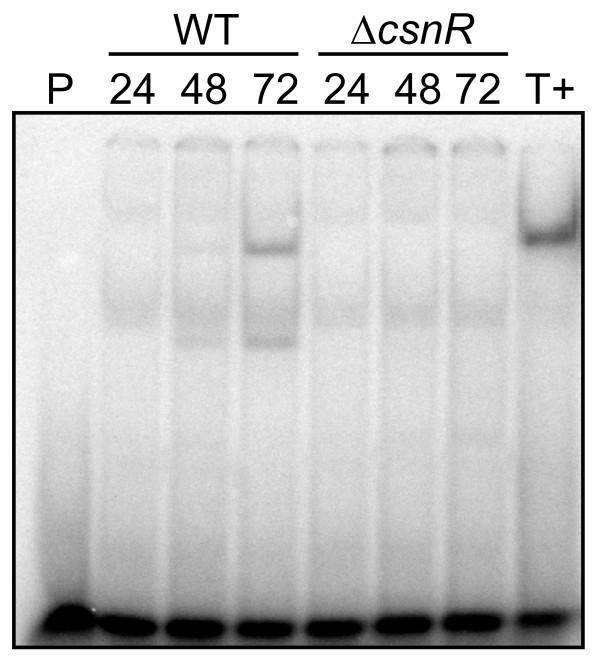
**Effect of *csnR *deletion on DNA-protein interaction at the *csnN106 *gene operator**. Gel retardation experiment was set up combining 0.1 nM double strand oligonucleotide probe covering the palindromic box of *csnN106 *with 10 μg of crude protein extracts from *S. lividans *TK24 strain **(WT) **or the *csnR *deleted strain **(*ΔcsnR*) **cultivated in medium with 0.125% GlcN and 0.375% chitosan oligomers for the time (hours) indicated. **P: **probe only; **T+: **control reaction with 2 μg of partially purified protein from *Kitasatospora *sp. N106 [39].

Crude extracts prepared from the cells of both strains cultivated in the presence of chitosan oligosaccharides (a mixture of GlcN and chitosan oligomer) were used in gel retardation experiments using a ^32^P-labelled oligonucleotide including the palindromic sequence from *csnN106 *as a probe. A shift in mobility was observed with the extract from the wild type strain but not with *ΔcsnR *mutant (Figure 3). The CsnR protein from *S. lividans *binds then efficiently the palindromic sequence of the heterologous *csnN106 *gene.

### Regulation of *csnN106 *chitosanase gene expression in the heterologous host

**Figure 4:**
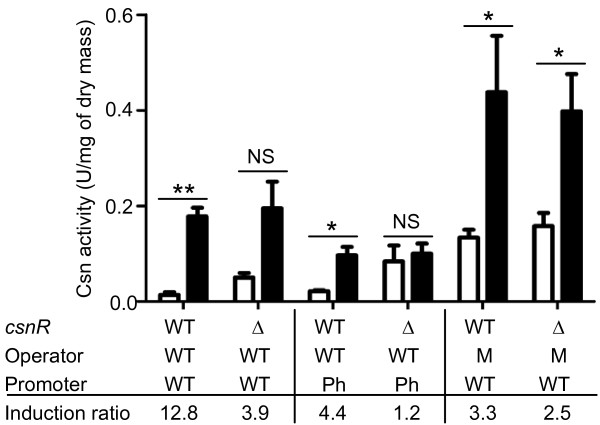
**Effect of mutations in *csnN106 *gene and *S. lividans *host on chitosanase production**. Chitosanase activity was assayed in supernatants sampled from 16 h cultures. Media: M14 M with 0.5% mannitol (empty columns) or M14 M with 0.125% GlcN and 0.375% chitosan oligomers (filled columns). Data and error bars are the mean of three experiments. ** P ≤ 0.01, * P ≤ 0.05 obtained with an unpaired t test (GraphPad Prism version 5.00 for Windows; GraphPad Software, San Diego, CA). The table lists the genotypes of strains for each pair of columns. Variants of *csnN106 *gene were introduced in one copy per genome *via *an integrative vector. Symbols: **WT: **wild type; **Δ: ***ΔcsnR *mutant host; **M: **mutated palindromic box; **Ph: **phage-type promoter. The induction ratio represents the chitosanase activity of culture induced with GlcN and chitosan oligomer divided by the activity of culture in mannitol medium.

To investigate the regulation of the *csnN106 *gene in *S. lividans*, various versions of the heterologous chitosanase gene have been cloned into derivatives of the integrative vector pHM8a. The wild-type and *ΔcsnR *strains of *S. lividans*, harbouring integrated variants of the *csnN106 *gene were cultivated in minimal media with either mannitol or a mix of chitosan oligosaccharides as carbon source. Samples of culture supernatants were collected after various incubation times and chitosanase activity and dry mycelia mass were measured. Figure 4 shows the chitosanase activities (expressed as Units/mg of dry mass to normalize with culture growth) attained after 16 h. The induction ratio is calculated dividing the activity obtained in the presence of chitosan oligosaccharides by that obtained in mannitol medium. Combining the host genotype, the chitosanase gene promoter and the palindromic sequence in their wild type forms resulted in the highest induction ratio (12.8x) indicating the extent of negative regulation of the *csnN106 *gene in the heterologous host. When the *csnR *gene was deleted or when the operator of *csnN106 *gene was mutated, the difference among activities produced in the presence and in the absence of chitosan became not significant or of low significance, resulting in low values of induction ratios (2.5 - 3.9), due essentially to derepression of chitosanase production in mannitol medium. The expression of the chitosanase gene with the phage-type promoter followed a similar pattern. Overall however, the phage-type promoter did not direct higher chitosanase production levels and was not included in further studies.

Globally, these results indicate that the palindromic sequence and the *csnR *gene function as a negative regulation system of the *csnN106 *gene in the heterologous *S. lividans *host. This mode of regulation is very similar to the one exerted on the endogenous chitosanase gene (*csnA*) of *S. lividans *TK24 (Dubeau *et al*., submitted).

### Chitosanase production in the absence of chitosan or derivatives

In our previous work, efficient production of chitosanase by either native or recombinant actinobacterial strains was strictly dependent on the addition of chitosan or derivatives (GlcN or chitooligosaccharides) in the culture media. The regulatory derepression, observed in the *S. lividans ΔcsnR *host harbouring the chitosanase gene with the mutated operator sequence raised the possibility to produce chitosanase in the absence of such inducers, using only inexpensive media components. Testing various concentrations of malt extract, salt formulations and methods of inoculation allowed obtaining routinely activities in the range of 10 - 12 units per ml and, in the best case, up to 24 units per ml (not shown). Protease activity was also highly dependent on medium composition and type of inoculum. Addition of magnesium ions was found to be essential to promote efficient chitosanase production (and low level of protease), while the microelements of the M14 M medium could be omitted (not shown).

**Figure 5:**
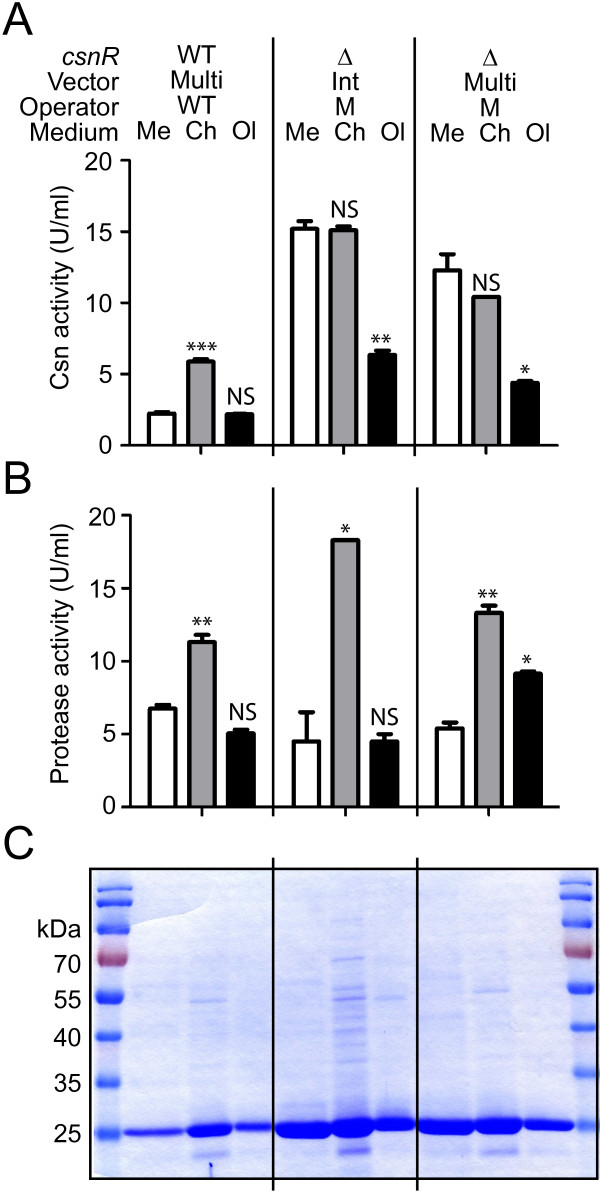
**Chitosanase activity and relative purity assessment and assay of protease levels**. **(A) **chitosanase activity; **(B) **protease activity; **(C) **SDS-PAGE of proteins in culture supernatants. The upper-table aligns the genotype of each strain and lists the type of medium for the corresponding columns in graphs **(A) **and **(B) **and wells of **(C)**. Genetic symbols as in Fig. 4. **Multi: **chitosanase genes introduced on a multi-copy vector; **Int: **chitosanase genes introduced on an integrative vector. Culture media: **Me: **malt extract medium; **Ch: **chitosan flakes medium; **Ol: **medium with GlcN and chitosan oligomers. All determinations have been done after 72 h of culture. Data and error bars **(A and B) **are the mean of culture duplicates. *** P ≤ 0.001, ** P ≤ 0.01, * P ≤ 0.05 from one-way ANOVA with Bonferroni's post test (GraphPad Prism version 5.00). **(C) 20 **μl of culture supernatants were loaded on a 12% SDS-PAGE gel. PageRuler™prestained protein ladder (0.5 μl; Fermentas) was used as standard. After electrophoresis, proteins were stained with Coomassie brilliant blue. Chitosanase migrates as a 26.5 kDa band.

In previous work, chitosanase production was performed with *S. lividans *TK24 harbouring *csn *genes originating from various bacterial species cloned in multicopy plasmids [35]. To compare the new gene/host combination with the former ones, we cloned the *csnN106 *gene (with a wild type operator) into the multicopy vector pFDES [28] and introduced it in the wild type host. In parallel, the same plasmid but with the mutated operator has been introduced into the *ΔcsnR *host. Chitosanase production by these two strains has been compared with that directed by the *csnN106 *gene (with the mutated operator) on a derivative of the integrative vector pHM8a in the *ΔcsnR *host. Three media formulations were tested: a medium containing malt extract as main nutrient source, a medium with chitosan flakes and GlcN, often used in our previous work, and a medium with more expensive components, GlcN and chitosan oligomers, used in basic research for the induction of chitosanase gene expression (Figure 4). On Figure 5 only the 72 h time point is presented, as chitosanase level was maximal around this time point and then remained stable or slightly decreased. The culture in medium with chitosan flakes and GlcN gives the best chitosanase level for the strain keeping intact both partners of the regulatory interaction (Figure 5A). However, cultures in media with chitosan gave much higher levels of extracellular proteases (Figure 5B). The *ΔcsnR *host harbouring the chitosanase gene on a integrative vector produced equivalent enzyme activities in the malt extract medium and in the chitosan flakes medium (Figure 5A), confirming the possibility to produce chitosanase in the absence of any chitosan derivative, with a much lower level of extracellular proteases (Figure 5B). Furthermore, the analysis of total extracellular proteins by SDS-PAGE revealed that there were less contaminant proteins in the malt extract medium than in the chitosan flakes medium (Figure 5C). In *ΔcsnR *host there was no particular advantage to use the multicopy plasmid over the integrative vector, the latter being more advantageous as it did not require the addition of any antibiotic to the medium. The *ΔcsnR *host seems to be particularly useful for the inexpensive production of almost pure chitosanase in stable, low-protease conditions.

## Discussion

This report is the first study dedicated to the genetic regulation of a heterologous chitosanase gene in *S. lividans*. We have shown that CsnR regulates negatively the expression of *csnN106 *gene. Deletion of *csnR *or mutations in the operator sequence of c*snN106 *resulted in the derepression of expression in the absence of inducer molecules. However, even in the derepressed gene/host combination, some residual induction by chitosan derivatives was still observed. This could be due to a regulator responding directly to the presence of chitosan or indirectly, through a stress pathway resulting from the interaction between chitosan and the cell. A complex transcriptomic response has been observed after contact with chitosan in cells of *Staphylococcus aureus *[41] and *Saccharomyces cerevisiae *[42].

One usual way to change the genetic regulation of a given gene is done by promoter replacement. In our earlier work, testing three different promoters from streptomycetes did not led to the improvement of chitosanase production [43]. In this work, we decided to replace only the -35 and -10 boxes from *csnN106 *promoter sequence while conserving all the remaining segments. Despite the use of a promoter considered as strong [38], this substitution did not result in better chitosanase production. For reasons that remain unclear, the chitosanase expression was less efficient for a total of four different hybrid gene constructions when the protein coding sequence of Csn was separated from its native upstream segment. This could result from a lower stability of mRNAs transcribed from these hybrid genes, but this remains to be investigated.

Masson *et al*. [35] optimized a chitosanase production medium for the CsnN174 production in the heterologous host *S. lividans*. They showed that the addition of malt extract to the chitosan medium was beneficial for enzyme production. We then based our media formulations on malt extract in our attempts to produce chitosanase with the new gene/host combination in the absence of chitosan. We have shown that equivalent, and sometimes higher chitosanase levels can be obtained without the addition of chitosan to the culture medium. Interestingly, the new medium/host combination resulted in much lower levels of contaminant proteins in the supernatant. Finally, in earlier culture media formulations including chitosan flakes, a raise of extracellular protease activity at later culture stage could often result in a rapid loss of chitosanase activity [35]. The new medium/host combination provides a substantial improvement, as protease levels are much lower, resulting in stable chitosanase production.

## Conclusions

The chitosanase production system based on a new medium/host combination was shown to be at least as efficient as the former one without the necessity to include chitosan or derivatives into the culture medium. Extensive optimization of culture parameters will probably lead to much higher chitosanase activities. For biotechnology, the new host will be of interest for large scale chitosanase production as only inexpensive media components can be used. For basic research, it will be particularly useful for the introduction of carbon or nitrogen isotopes into the chitosanase molecule, originating from defined sources such as ^13^C-glucose or ^15^NH_4_Cl and for the production of highly pure chitosanase proteins for crystallography. This will contribute to a further advance in structure-function studies of chitosanases.

## Methods

### Bacterial strains and general culture conditions

*E. coli *strain DH5α™(Invitrogen) was used for cloning experiments and DNA propagation. *E. coli *DH5α™was grown on Luria-Bertani broth supplemented with 500 μg/ml hygromycin (Hm) or 50 μg/ml kanamycin (Km). Standard methods were used for *E. coli *transformation, plasmid isolation and DNA manipulation [44]. *Streptomyces lividans *TK24 [45] and *S. lividans ΔcsnR *[37] were used as hosts for chitosanase genes. Preparation of *S. lividans *protoplasts and transformation using rapid small-scale procedure and R5 regeneration medium were performed as described previously [45]. After DNA transfer, hygromycin or kanamycin-resistant colonies were selected after addition of 5 mg Hm or Km to 2.5 ml of soft agar overlay. Transformants were chosen following two subsequent cycles of purification on solid yeast/malt extract (YME) medium [45] with 250 μg/ml Hm or Km. Sporulation was obtained by heavy inoculation of SLM3 agar medium plates [46]. Spores were collected with glass beads and stored in 20% glycerol at -20°C.

### Gel mobility shift assay

10^8 ^spores of *S. lividans *TK24 or *S. lividans ΔcsnR *were inoculated into 50 ml of Tryptic soy broth (TSB, Difco) and grown for 64 h at 30°C with shaking. Cultures were centrifuged, the mycelial pellets were washed with sterile 0.9% saline and suspended in two volumes of saline. Then, 1 mpv (equivalent of 1 ml of pellet volume) was added to 100 ml of induction medium. Induction medium is a modified M14 medium (M14M) [47] composed of 0.1% KH_2_PO_4_, 0.55% K_2_HPO_4_, 0.14% (NH_4_)_2_SO_4_, 0.1% of trace elements solution (2 g/L CoCl_2_·7H_2_O, 5 g/L FeSO_4_·7H_2_O, 1.6 g/L MnSO_4_·H_2_O, 1.4 g/L ZnSO_4_·7H_2_O), pH 6.9. Before use, 0.03% MgSO_4_, 0.03% CaCl_2_, 0.125% GlcN and 0.375% chitosan oligomers (1:1 dimer-trimer mix) was added to the M14 M. Cultures were incubated at 30°C with shaking. Every 24 h, 10 ml of culture were collected and centrifuged and pellets were kept frozen at -80°C. Pellets were melted on ice, washed with cold extraction buffer (50 mM Tris, 60 mM NaCl, 5% glycerol, 1 mM EDTA, 1 mM DL-dithiothreitol (DTT), pH 8.0) and suspended in 1 ml of extraction buffer containing a protease inhibitor cocktail (Complete™; Roche Molecular Biochemicals). The bacterial cells were then disrupted by sonication with one treatment of 40 s at 40% amplitude (Vibra-Cell™, 130 Watt 20 kHz, Sonics and materials inc., USA). Total protein extracts were centrifuged at 3000 g for 10 min at 4°C. Supernatants were then frozen and stored at -80°C until used.

The double-stranded *csnN106 *palindromic probe (MP12F) was prepared by complementary oligonucleotide annealing and end-labeling with [γ-^32^P]ATP (PerkinElmer) and T4 polynucleotide kinase as described by Dubeau *et al*. [36]. DNA binding reactions (24 μl) contained 10 mM HEPES (pH 7.9), 10% glycerol, 0.2 mM EDTA, 0.5 mM PMSF, 0.25 mM DTT, 1 μg poly(dI-dC), 150 mM KCl, 0.1 nM of labeled probe and 10 μg of protein crude extract. The reaction mixtures were incubated at room temperature for 15 min and then subjected to electrophoresis in a pre-run gel of 6% polyacrylamide (10 mM Tris, 80 mM glycine, 0.4 mM EDTA, pH 8.3). The gel was dried and viewed with a Phosphorimager (Molecular Dynamics).

### Vector construction

The *csnN106 *gene fragment (GenBank accession number L40408.1) was amplified by PCR reaction using Fw*csnN106 *and Rv*csnN106 *primers (Table 1) and plasmid pCSN106-2 as template [32]. The amplified *Sph*I - *Hin*dIII fragment was cloned into the integrative vector pHM8aBΔM [37] or pFDES [28] digested with the same enzymes, giving respectively plasmids pHM8aBΔM-*csnN106 *and pFDES-*csnN106*. The promoter region of *csnN106 *(Pr-WT) was PCR-amplified with primers FwPr-WT and RvPr-WT. Purified PCR fragment was cloned between *Bam*HI and *Sph*I restriction sites of pHM8aBΔM-*csnN106 *and pFDES-*csnN106 *generating pHPr-WT and pFPr-WT. A mutated version of Pr-WT with two base-pairs substitutions in the palindromic operator (Pr-Pa) was obtained with the PCR-directed mutagenesis method [48] using SEQ.1, Rv1Pr-Pa, Fw2Pr-Pa and Rv*csnN106 *as primers (Table 1) and the pFPr-WT plasmid as DNA template. The mutated PCR product was digested with *Bam*HI and *Sph*I and cloned into pHM8aBΔM-*csnN106 *and pFDES-*csnN106 *generating pHPr-Pa and pFPr-Pa. The phage-type version of *csnN106 *promoter (Pr-Ph) was obtained by annealing two short DNA segments:

5'-GATCCTGACGGCCCGTCCGCCCAGCGGTACGAGGGCCCCGACCGGAGTTCCGGTCGGGGCCTTTCGCATGACCGCGCGGGCAAACAT

GGCGCTTGACCTTGATGAGGCGGCGTGAGCTACAATCAATATCTAGTTAGGAAACTTTCCTAACTCTCCTCATGGGTCCGGAGACCCGCATG-3'

 and 5'-CGGGTCTCCGGACCCATGAGGAGAGTTAGGAAAGTTTCCTAACTAGATATTGATTGTAGCTCACGCCGCCTCATCAAGGTCAAGC

GCCATGTTTGCCCGCGCGGTCATGCGAAAGGCCCCGACCGGAACTCCGGTCGGGGCCCTCGTACCGCTGGGCGGACGGGCCGTCAG-3'.

 As a result, the double-stranded oligonucleotide with "ready-to-clone" cohesive-ends was ligated with pHM8aBΔM-*csnN106 *digested with *Bam*HI and *Sph*I generating pHPr-Ph. All constructions were verified by DNA sequencing (Genome Quebec Innovation Center, McGill University, Canada).

**Table 1:** Oligonucleotides used in this study

Aim of primers	Name	Sequence (5'→3')
For ***csnN106 ***coding region cloning*	Fw***csnN106***	CCGGAGACCC**GCATGC**CCCGGAC
	Rv***csnN106***	CGGTGCGCC**AAGCTT**GCGTTCGG

For Pr-WT cloning*	FwPr-WT	GTCTGCGC**GGATCC**TGACGGCCC
	RvPr-WT	GTCCGGG**GCATGC**GGGTCTCCGG

PCR-directed mutagenesis for Pr-Pa cloning**	SEQ.1	ACAACTTCGTCGCGCACATCCA
	Rw1Pr-Pa	ATGAGGAGAGTTCGGACAGTTTC
	Fw2Pr-Pa	GAAACT**G**TCC**G**AACTCTCCTCAT
	Rv***csnN106***	TGAGGTCGAAGTTCTTGGCGTT

Verification of pHM8a derivatives integration into hosts	Fwgenom	CCTGAGAGGCCGGTGAGGAG
	Rv***csnN106***	TGAGGTCGAAGTTCTTGGCGTT

Presence verification of pFDES derivatives into hosts	SEQ.1	ACAACTTCGTCGCGCACATCCA
	T7 promoter	TTAATACGACTCACTATAGGG

For Primer extension	PE-***csnN106***	TGGGGTGCTTGAGACGCAT

*Bold nucleotides correspond to restriction site

**Bold nucleotide correspond to mutated nucleotide

Plasmids were introduced into *S. lividans *strains by transformation and selection with Hm for pHM8a derivatives carrying *hyg *or selection with Km for pFDES derivatives carrying *neoS *as resistance gene. Integration of pHM8a derivatives into the genome of their hosts and the presence of pFDES derivatives were verified by PCR using primers in Table 1.

### Transcription startpoint mapping by primer extension

10^8 ^spores of *S. lividans *TK24(pHPr-WT) strain were inoculated into 50 ml of TSB with 50 μg/ml Hm and grown for 64 h at 30°C with shaking. Chitosanase gene expression was obtained in M14 M medium with GlcN and chitosan oligomers as described for gel mobility shift assay. After 14 h, four culture samples of 10 ml each were collected and mixed immediately with stop solution (0.2 volumes of ethanol-equilibrated phenol, 95:5). Samples were centrifuged for 10 min at 4°C. Bacterial pellets were frozen at -80°C until lysis. Total RNA extraction was carried out using the Qiagen RNeasy^® ^Mini Kit (Qiagen) with the following modifications. Cell disruption was achieved by sonication with two 30 s burst at 35% amplitude separated with a 15 s cooling period, followed by two phenol-chloroform extractions and one chloroform extraction for cell debris elimination. The on-column DNase treatment was done with the RNase-free DNase set (Qiagen). RNA purity and concentration were assessed in a NanoDrop™1000 spectrophotometer (Thermo Scientific). RNA quality was verified by electrophoresis on agarose gel in 1× MOPS electrophoresis buffer with 0.22 M formaldehyde [44].

20 ρmoles of PE-*csnN106 *primer (Table 1) were end-labeled with [γ-^32^P]ATP (PerkinElmer) and 20 units of T4 polynucleotide kinase, then purified on a G-25 column (GE Healthcare). Total RNA (40 μg) was hybridized with the end-labeled primer (0.5 ρmole) in the presence of 10 mM Tris-HCl pH 8.6, 300 mM NaCl and 1 mM EDTA, in a volume of 22 μl by incubation at 95°C for 5 min, then 55°C for 90 min. ARN/primer mix was then precipitated with 200 μl ammonium acetate 1 M and 200 μl isopropanol. The pellet was washed with 70% EtOH, dried and suspended in 10 μl of 10 mM Tris-HCl (pH 8.6), reverse transcriptase buffer (1×, Promega), 10 mM DTT, 1 mM dNTPs, 1 μg actinomycine D, 5 units of AMV reverse transcriptase (Promega) and 20 units of RNAsin (Promega) for a total volume of 20 μl. The reaction mixture was incubated at 45°C for 60 min and stopped with formamide dye. A sequencing reaction was performed with the end-labeled primer, the pHPr-WT plasmid as DNA template and the ALFexpress™AutoCycle™Sequencing Kit (Amersham Biosciences) using manufacturer's recommendations. The primer extension sample and the sequence reactions were heated 5 min at 95°C just before loading on a 6% polyacrylamide sequencing gel. The gel was run, dried, visualised and analyzed by a Phosphorimager and the ImageQuant Version 5.2 software (Molecular Dynamics).

### Chitosanase production experiments

A first procedure was used for experiments presented on Figure 4. 10^8 ^spores of *S. lividans *strains (transformed with plasmids pHPr-WT, pHPr-Pa or pHPr-Ph) were inoculated into 50 ml of TSB with 50 μg/ml Hm and grown for 48 h at 30°C with shaking. After centrifugation, bacterial pellets were transferred into M14 M with 0.125% GlcN and 0.375% chitosan oligomers (induction medium) or 0.5% mannitol (control medium), as described for gel mobility shift experiments. Samples of cultures of 10 ml each were collected at 12 h, 16 h, 22 h and 38 h and centrifuged. Chitosanase activity was determined in the culture supernatants, while pellets were used for dry weight measurement determination, drying overnight at 50°C.

A second procedure was used for the experiments presented on Figure 5. 10^9 ^spores of *S. lividans *strains (WT + pFPr-WT, *ΔcsnR *+ pHPr-Pa, *ΔcsnR *+ pFPr-Pa) were inoculated into 50 ml of TSB supplemented with 50 μg/ml Km (WT + pFPr-WT and *ΔcsnR *+ pFPr-Pa) or 50 μg/ml Hm (*ΔcsnR *+ pHPr-Pa) and grown for 64 h at 30°C with shaking. Three types of culture were tested. First, a rich, malt extract-based medium (4× M14 M without microelements, 0.12% MgSO_4_, 2% malt extract) was directly inoculated with a portion of the pre-culture in TSB corresponding to an inoculation proportion of 4 mpv/100 ml. Second, 100 ml of chitosan medium (M14 M, 0.03% MgSO_4_, 0.03% CaCl_2_, 0.2% malt extract, 0.8% chitosan flakes (Sigma), 0.2% GlcN) was inoculated with 1 mpv of saline washed pre-culture. Third, 100 ml of GlcN/chitosan oligomer medium (M14 M, 0.03% MgSO_4_, 0.03% CaCl_2_, 0.125% GlcN and 0.375% chitosan oligomers) was inoculated with 1 mpv of saline washed pre-culture. For each WT + pFPr-WT and *ΔcsnR *+ pFPr-Pa flasks, 50 μg/ml Km was added. Cultures were done in duplicate and incubated at 30°C with shaking. 10 ml samples were collected every 24 h. Chitosanase and protease activities and total protein concentration were determined in supernatants.

### Biochemical procedures

Chitosanase activity was measured using the dyed substrate sRBB-C [49]. Briefly, 50 μl of appropriately diluted culture supernatant were added to 950 μl of soluble Remazol Brilliant Blue chitosan (5 mg/ml in 0.1 M Na-acetate buffer pH 4.5) and the mixture was incubated for 60 min at 37°C. Reaction was stopped with 500 μl of 1.2 N NaOH and cooled on ice for 20 min. After centrifugation, the optical density of supernatant was read at 595 nm and converted into chitosanase activity as described [49].

Protein concentration was estimated by the method of Bradford [50], with bovine serum albumin as standard. Protease activity was determined with azocasein [51].

## Competing interests

The authors declare that they have no competing interests.

## Authors' contributions

RB and M-PD initiated and coordinated the project. RB performed the bioinformatic studies. M-PD performed the plasmids and strains constructions and the DNA retardation experiments. IG and M-PD performed the induction experiments. M-PD and RB wrote the article and all authors approved the final version of the manuscript.
